# Parental Somatic Mosaicism Uncovers Inheritance of an Apparently *De Novo GFAP* Mutation 

**DOI:** 10.3389/fgene.2021.744068

**Published:** 2021-12-07

**Authors:** Alice Grossi, Federico Morelli, Marco Di Duca, Francesco Caroli, Isabella Moroni, Davide Tonduti, Tiziana Bachetti, Isabella Ceccherini

**Affiliations:** ^1^ UOSD Laboratory of Genetics and Genomics of Rare Diseases, IRCCS Istituto Giannina Gaslini, Genoa, Italy; ^2^ Laboratory of Molecular Nephrology, IRCCS Istituto Giannina Gaslini, Genoa, Italy; ^3^ Department of Pediatric Neurosciences, Fondazione IRCCS Istituto Neurologico Carlo Besta, Milan, Italy; ^4^ Unit of Pediatric Neurology - C.O.A.L.A (Center for Diagnosis and Treatment of Leukodystrophies), V. Buzzi Children’s Hospital, Milan, Italy; ^5^ Laboratory of Developmental Neuro-Biology, DISTAV, University of Genoa, Genoa, Italy

**Keywords:** central nervous system diseases, genetic counseling, human genetics, DNA sequence analysis, *GFAP* gene, Alexander disease, germline mosaicism, somatic mosaicism

## Abstract

Alexander disease is a leukodystrophy caused by heterozygous mutations of *GFAP* gene. Recurrence in siblings from healthy parents provides a confirmation to the transmission of variants through germinal mosaicism. With the use of DNA isolated from peripheral blood, next-generation sequencing (NGS) of *GFAP* locus was performed with deep coverage (≥500×) in 11 probands and their parents (trios) with probands heterozygous for apparently *de novo GFAP* mutations. Indeed, one parent had somatic mosaicism, estimated in the range of 8.9%–16%, for the mutant allele transmitted to the affected sibling. Parental germline mosaicism deserves attention, as it is critical in assessing the risk of recurrence in families with Alexander disease.

## Introduction

Alexander disease (AxD) is an extremely rare, untreatable, and usually fatal neurodegenerative disorder (OMIM #203450), classified among leukodystrophies due to white matter deficits (Messing and Brenner, 2020). It is estimated to affect 1:2.7 million people in Japan (Yoshida et al., 2011). The disease presents at different ages of onset, with distinct symptoms and prognosis: in neonates and early childhood (type I) and later, though not restricted to adulthood (type II) (Prust et al., 2011). AxD is caused by heterozygous mutations of glial fibrillary acidic protein *(GFAP)* gene, which eventually lead to the formation of aggregates, also containing alphaB-crystallin, HSP27, ubiquitin, and proteasome components (Quinlan et al., 2007). To date, a broad spectrum of pathogenic *GFAP* variants accounts for more than 90% of patients. Mutations occur either de novo or through transmission from the parental generation. A recurrent occurrence of the same disease-causing GFAP mutation in siblings from parents who tested negative for the variant strongly suggests the presence of a germinal mosaicism (Melchionda et al., 2013) (two affected siblings were also reported by Namekawa et al. (2002), but the parents were not examined). Indirect evidence for germinal mosaicism in de novo AxD cases has also been provided by studies finding that the *de novo* mutations predominantly arise on the paternal chromosome (Li et al., 2006; Zang et al., 2013). Such a condition may be associated with somatic mosaicism, a circumstance nevertheless unproven so far (Messing, 2018). In the case of AxD, the risk of transmitting a *GFAP* mutation to a second child by germline mosaicism has been estimated as less than 1% (Messing, 2018); however, when significant somatic mosaicism is observed in a parent, the risk of recurrence could be substantially higher.

In the initial diagnostic screening of patients affected with AxD, and their parents when available, the entire coding region of *GFAP* gene was examined by Sanger sequencing. To test the hypothesis that during our diagnostic workflow the mosaicism of somatic *GFAP* mutations in AxD families may have been under-recognized, 11 pairs of asymptomatic parents of AxD patients heterozygous for *GFAP* causative and apparently *de novo* mutations were selected and then subjected to targeted in-depth sequencing.

## Materials and Methods

As the next-generation sequencing (NGS) technology can facilitate the detection of low-level mosaicism, undetectable by conventional Sanger sequencing (Qin et al., 2016), deep coverage (≥500×) NGS, targeting *GFAP* locus, was therefore performed, as already described (Rusmini et al., 2016), using DNA isolated from peripheral blood samples from 11 pairs of asymptomatic parents without previously detected *GFAP* mutations, whose siblings are affected with AxD and heterozygous for a causative *GFAP* mutation already detected in the past by Sanger sequencing. In particular, the samples to be re-evaluated were just plugged into a new diagnostic pipeline making use of an NGS-based gene panel setup more recently.

The genomic region included in the experimental design and sequenced is at chr17:4,2,982,001–42,997,000 (GRCh37/hg19), contains regulatory regions and UTRs, all nine exons, and flanking regions (GFAP reference transcript NM_002,055.5), is divided into 59 amplimers of 264 bases on average and has a final coverage of 95%, with 752 bases missing, mostly intronic. Our attention was focused on two amplimers of *GFAP* gene containing variants under analysis, namely, p.E75K, p.L76P, p.R79C/H (chr17:42,992,581–42,992,886, according to GRCh37 and corresponding to exon 1), and p.R239C (chr17:42,990,593–42,990,894, according to GRCh37 and corresponding to exon 4). The parameters for the variant calling were configured in the custom mode recommended for detecting mosaicisms (Torrent Suite Software, Thermo Fisher^®^, Thermo Fisher Scientific, Waltham, MA, USA).

Single-nucleotide primer extension was performed using a specific kit (SNaPshot Multiplex Sistem, Thermo Fisher^®^) and oligos suitable for the mutation site (p.R79C) on both strand (forward primer: 5′-GAG​ATG​ATG​GAG​CTC​AAT​GAC​C-3; reverse primer: 5′-CTT​CTC​GAT​GTA​GCT​GGC​AAA​G-3′), followed by capillary electrophoresis on automated DNA sequencer (ABI PRISM^®^ 3130XL), according to a protocol already reported (Matera et al., 2013).

The PCR product of exon 1 was cloned using the genomic DNA of ID#110M subject as a template, into the TOPO TA vector (Invitrogen, Carlsbad, CA, USA) following the manufacturer’s instructions. The plasmid DNA isolated from 51 clones was directly sequenced using the Sanger protocol, as already reported (Matera et al., 2013).

## Results

Supplementary Table S1 reports details on the in-depth NGS analysis of AxD-causing GFAP mutations in families with apparently *de novo* occurrence, all representing single instances of sibs having a mutation. In particular, the coverage of the amplicons containing the mutant codons is reported for each indicated patient’s parents (mother or father). All parents could be evaluated for possible mosaicism at the mutation site of corresponding patients, except the father of proband #147 (ID#147F), who had to be removed from the set because the coverage of the region, affected by the mutation present in his son, did not reach the minimum threshold established by the experimental design (500×).

**FIGURE 1:**
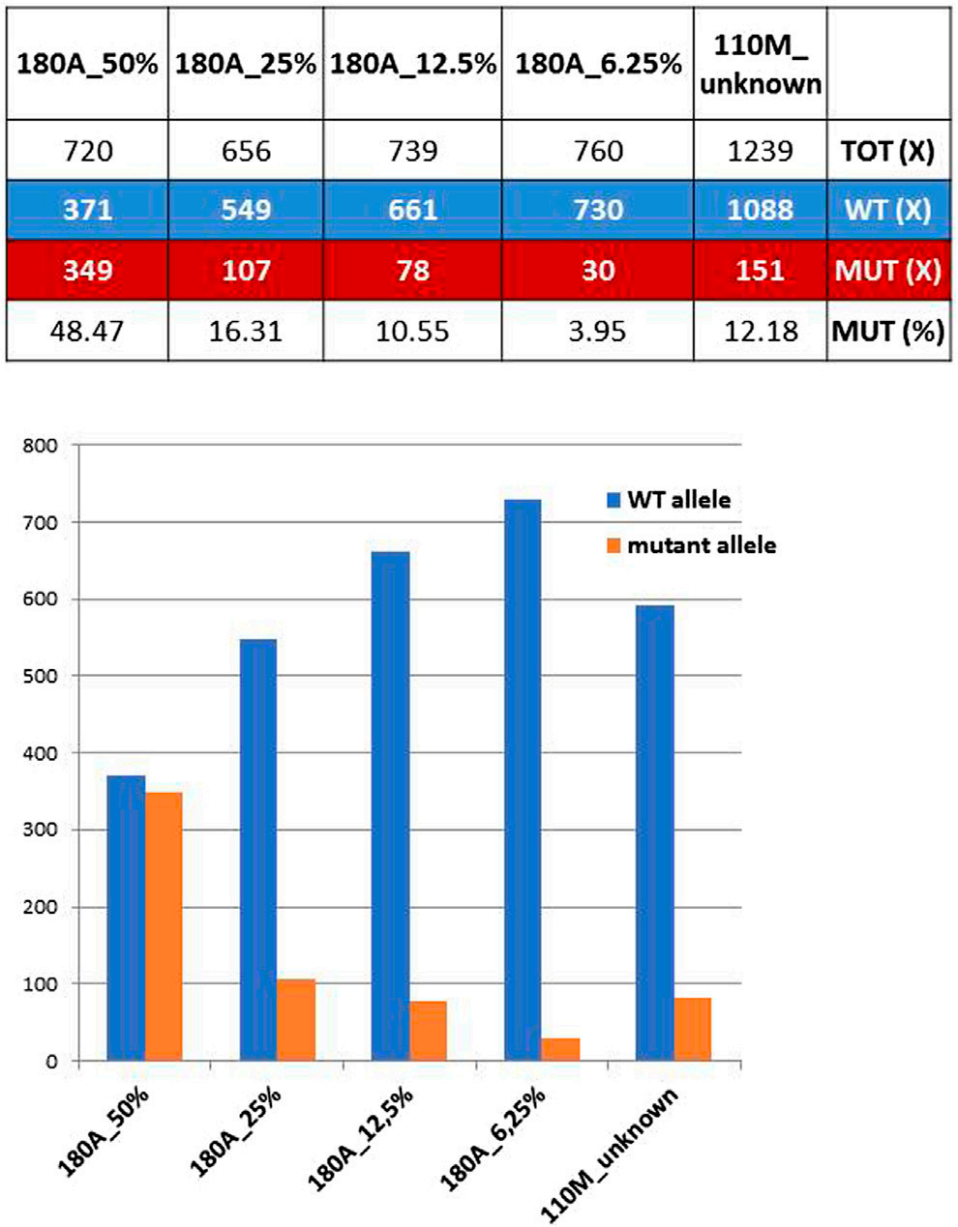
Coverage ratios for mutant and wild-type (WT) alleles obtained from known dilutions of a defined heterozygous control (ID#180A) are compared with those of ID#110M parent. Bottom: WT and mutant allele reads shown above are reported as bars of a histogram.

The mother of proband #110 (ID#110M) revealed a mosaicism of *GFAP* mutation already detected in her son, the c.236G>A (p.R79H). The ratios between mutant and total allele reads amount to 12.18%, which can be regarded as the extension of the mosaicism in blood cells for this mother. However, when the 12.18% value was compared with ratios obtained previously with known dilutions of the mutant allele of a heterozygous control, ID#180A (the patient of family #180) carrying the mutations *GFAP* c.227T>C (p.L76P), an estimate of the extension of mosaicism of 16% was achieved (Figure 1), a figure higher than expected and likely affected by technical biases and experimental variability.

**FIGURE 2:**
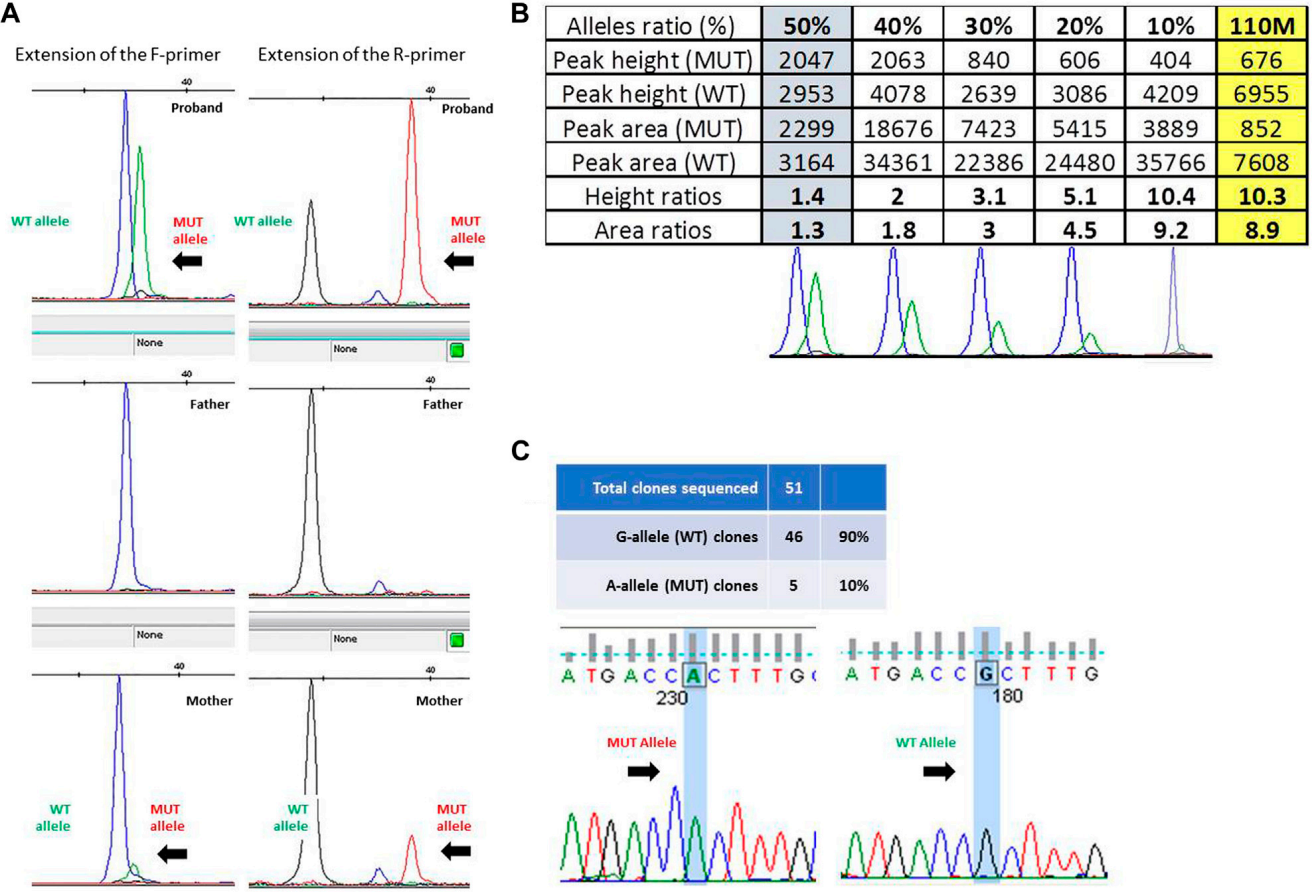
**(A)** Fluorescence peak of the labeled nucleotide signal inserted at the mutation site, obtained with forward (F) and reverse (R) primers in trio ID#110. **(B)** Peak height and area ratios obtained from ID#110M DNA sample (last yellow column) with bottom reference curves constructed using DNA mixtures containing known fractions of the two alleles, i.e., 50%, 40%, 30%, 20%, and 10% from left to right. Given that results from both strands were very consistent, to optimize the figure and to avoid redundant information, only data obtained from the “F” primer are ultimately reported here. **(C)** Electropherogram of the sequences obtained from colonies of *Escherichia coli* with wild-type and mutated alleles and ratio between the number of colonies obtained.

The presence of a small proportion of mutant allele was confirmed through the single-nucleotide primer extension approach, evaluating the amount of the wild-type and mutant alleles obtained from ID#110 families by using both the F- and R-primers (Figure 2A) and then comparing the ratios of peak height and of peak area obtained from ID#110M DNA sample (right-hand yellow column) with a reference curve constructed using DNA mixtures containing known fractions of the two alleles (Figure 2B). This allowed us to estimate an 8.9% proportion of mutant allele in this mosaic mother.

Given the low and uncertain proportion of the expanded alleles in this latter parent, we sought to confirm her mosaic status by cloning the PCR product of exon 1, containing *GFAP* mutation c.236G>A (p.R79H). Overall, 51 colonies were obtained, 46 of which showed the presence of a wild-type G allele at the mutation site after Sanger sequencing. This allowed us to estimate a ratio of clones carrying mutant and wild-type inserts of 10% (Figure 2C).

Based on the different approaches applied and the comparably similar estimates obtained (8.9%, 10%, 12.18%, and 16%), it could therefore be concluded that ID#110M mother shows a rather low degree of mosaicism, although not irrelevant for *GFAP* mutation c.236G>A (p.R79H).

A retrospective reanalysis, carried out on the Sanger sequences initially obtained from this mother’s DNA, showed a very small second/mutant peak allele underneath the reference allele, which however was not attributed to the mutant allele but rather to the background noise (data not shown).

## Discussion

Somatic mosaicism, occurring as a result of postzygotic mutations, can affect human health and diseases (Acuna-Hidalgo et al., 2015; D’Gama and Walsh, 2018). Indeed, as far as AxD is concerned, one patient was reported as a somatic mosaic for a *GFAP* mutation that was detected in buccal DNA (presumably present also in brain), but not in blood DNA, clearly arising in the embryo rather than in the parental germline (Flint et al., 2012). Moreover, the combination of both somatic and germline mosaicisms has been postulated to account also for another mechanism recently suggested in AxD, where the presence of a *GFAP* mutation only in a subset of cells in the parental generation could attenuate disease severity and delay the age of onset, thus leading to an apparent anticipation of symptoms in the offspring expressing the mutation ubiquitously. Unfortunately, authors have been unable to test this hypothesis that should be considered anyway in future instances of inherited AxD (Hunt et al., 2021). Finally, rare repetitive occurrences of AxD were already described in families, in association with severe GFAP mutations likely transmitted from parents who did not have symptoms and/or had tested negative for the mutation; however, no germline mosaicism has yet been demonstrated (Namekawa et al., 2002; Melchionda et al., 2013). Mosaic mutations in parents, and particularly mosaicism due to mutations that occurred at an earlier stage and therefore present in both the germline and somatic cells, could underlie a proportion of these cases, otherwise attributable to *de novo* germline mutations. Here, we have confirmed AxD transmission through germline mosaic *GFAP* mutation, made evident by the demonstration of somatic mosaicism, a circumstance that requires specific and careful workout especially for rare diseases, as already described (Bachetti et al., 2011; Brewer et al., 2020).

Recognition of a mosaicism can be achieved with different methods, each with a different resolution. A major obstacle to observing mosaicism is that the testing sample should harbor the mutation, given the surprisingly variable levels of mosaicism potentially detectable between tissues and organs and even within the same embryonic lineages (Campbell et al., 2015).

A roughly 12% mosaic condition for *GFAP* c.236G>A (p.R79H) mutation, a genetic defect already shown in association with AxD and recurring in unrelated patients (Rodriguez et al., 2001; Asahina et al., 2006; Zang et al., 2013), has been demonstrated in blood cells of an AxD patient’s mother, and for this reason, it must have been present to an unknown extent in the lineage of the germ cells. Unfortunately, we did not have the opportunity to test DNA from tissues other than whole blood, such as exfoliation cells found in oral mucosa swabs, urinary sediments, skin hair bulbs, or other tissues, to investigate the extent of somatic mosaicism. Depending on when the somatic mutation occurs, we expect a different involvement of different tissues that can carry heterogeneous cell populations together with tissues characterized instead by a homogeneous wild-type cell population. This is also consistent with the possible onset of the disease phenotype, whenever mutant cells are found within tissues that can be affected by the presence of the mutation. Subject ID#110M, identified as a mosaic in the present study, is asymptomatic, and this can be explained by postulating that the mutation is absent in the astrocyte cell line of the central nervous system, the target of the neurodegeneration typical of AxD and in which *GFAP* is expressed. Nevertheless, both the presence of the mosaicism at a tolerable low level in astrocytes and a late onset of a mild disease cannot be ruled out at this stage.

Finding a *GFAP* mutation in an AxD patient and following its segregation within the family is crucial both to confirm a diagnosis and to assess the risk of disease recurrence. Actually, families with *de novo* mutations are generally considered as at risk for possible germline mosaicism and should be counseled accordingly. Given the high probability of germline mosaicism (Li et al., 2006), and indeed genetic counseling and possibly prenatal testing during a second pregnancy are already recommended for families at risk of AxD recurrence, the discovery that the patient’s mother ID#110M is actually a mosaic has represented a confirmation for parents of their actual transmission hazard. Indeed, the discovery of germline mosaicism exposes this family to a much higher but unquantifiable risk of AxD recurrence: though the possibility that 100% of oocyte cells carrying this variant cannot be excluded, the real risk of recurrence in this family is presumably lower than the 50% risk typical of germline carriers (Gajecka, 2016; Machiela and Chanock, 2017).

Although the search for parental somatic, and therefore germline, mosaicisms should make use, whenever possible, of tissues relevant to the disease, NGS with deep coverage at target sites of even less appropriate DNA sources may represent a fruitful approach to this type of investigation. Obviously, the test becomes informative only if a somatic mutation is found, while for subjects with negative results, mosaicism cannot be definitely excluded (Gajecka, 2016; Machiela and Chanock, 2017).

The tools provided by NGS, and in particular the possibility of an in-depth analysis, allow for the detection of possible mosaicisms, thus providing safer and more informative genetic counseling, an opportunity that should be considered for many rare diseases, including AxD.

## Data Availability

The original contributions presented in the study are all included in the article. The novel observation of the p.R79H GFAP variant found in a mosaic parent is deposited in the ClinVar database and it has been assigned ID = SCV001786708. Further inquiries can be directed to the corresponding author.
