# Corrigendum: Parental Somatic Mosaicism Uncovers Inheritance of an Apparently De Novo GFAP Mutation

**DOI:** 10.3389/fgene.2022.877443

**Published:** 2022-03-21

**Authors:** Alice Grossi, Federico Morelli, Marco Di Duca, Francesco Caroli, Isabella Moroni, Davide Tonduti, Tiziana Bachetti, Isabella Ceccherini

**Affiliations:** ^1^ UOSD Laboratory of Genetics and Genomics of Rare Diseases, IRCCS Istituto Giannina Gaslini, Genoa, Italy; ^2^ Laboratory of Molecular Nephrology, IRCCS Istituto Giannina Gaslini, Genoa, Italy; ^3^ Department of Pediatric Neurosciences, Fondazione IRCCS Istituto Neurologico Carlo Besta, Milan, Italy; ^4^ Unit of Pediatric Neurology - C.O.A.L.A (Center for Diagnosis and Treatment of Leukodystrophies), V. Buzzi Children’s Hospital, Milan, Italy; ^5^ Laboratory of Developmental Neuro-Biology, DISTAV, University of Genoa, Genoa, Italy

**Keywords:** central nervous system diseases, genetic counseling, human genetics, DNA sequence analysis, germline mosaicism, somatic mosaicism, GFAP gene, Alexander disease

In the original article, there was an error in the **Introduction** section. The phrase “siblings of parents” seems to imply the aunts and uncles of the affected child, rather than the siblings of the child, as it should be. A correction has been made in the **Introduction** section:

Alexander disease (AxD) is an extremely rare, untreatable, and usually fatal neurodegenerative disorder (OMIM #203450), classified among leukodystrophies due to white matter deficits (Messing and Brenner, 2020). It is estimated to affect 1:2.7 million people in Japan (Yoshida et al., 2011). The disease presents at different ages of onset, with distinct symptoms and prognosis: in neonates and early childhood (type I) and later, though not restricted to adulthood (type II) (Prust et al., 2011). AxD is caused by heterozygous mutations of glial fibrillary acidic protein (*GFAP*) gene, which eventually lead to the formation of aggregates, also containing alphaB-crystallin, HSP27, ubiquitin, and proteasome components (Quinlan et al., 2007). To date, a broad spectrum of pathogenic *GFAP* variants accounts for more than 90% of patients. Mutations occur either *de novo* or through transmission from the parental generation. A recurrent occurrence of the same disease-causing GFAP mutation in siblings from parents who tested negative for the variant strongly suggests the presence of a germinal mosaicism (Melchionda et al., 2013) (two affected siblings were also reported by Namekawa et al. (2002), but the parents were not examined). Indirect evidence for germinal mosaicism in *de novo* AxD cases has also been provided by studies finding that the *de novo* mutations predominantly arise on the paternal chromosome (Li et al., 2006; Zang et al., 2013). Such a condition may be associated with somatic mosaicism, a circumstance nevertheless unproven so far (Messing, 2018). In the case of AxD, the risk of transmitting a *GFAP* mutation to a second child by germline mosaicism has been estimated as less than 1% (Messing, 2018); however, when significant somatic mosaicism is observed in a parent, the risk of recurrence could be substantially higher.

Also, a reference “**Li et al., 2006” was cited but** was not included in the reference section. A correction has been made to the **Reference** list.

The authors apologize for this error and state that this does not change the scientific conclusions of the article in any way. The original article has been updated.

## References

[B1] Li R. Johnson A. B. Salomons G. S. van der Knaap M. S. Rodriguez D. Boespflug-Tanguy O. (2006). Propensity for Paternal Inheritance of *De Novo* Mutations in Alexander Disease. Hum. Genet. 119 (1–2), 137–144. 10.1007/s00439-005-0116-7 16365765

